# Epigallocatechin-3-gallate and cancer: focus on the role of microRNAs

**DOI:** 10.1186/s12935-023-03081-8

**Published:** 2023-10-14

**Authors:** Chunguang Wang, Meiling Bai, Zhiguang Sun, Nan Yao, Aiting Zhang, Shengyu Guo, Zatollah Asemi

**Affiliations:** 1 The First Affiliated Hospital of Hebei North University, Zhang Jiakou, 075000 Hebei China; 2 Basic Medical College of Hebei North University, Zhang Jiakou, 075000 Hebei China; 3 Research Center for Biochemistry and Nutrition in Metabolic Diseases, Institute for Basic Sciences, Kashan University of Medical Sciences, Kashan, Islamic Republic of Iran

**Keywords:** Cancer, Epigallocatechin-3-gallate, Epigenetic, MicroRNAs, Molecular pathways

## Abstract

MicroRNAs (miRNAs) are a group of small non-coding RNAs that affect gene expression. The role of miRNAs in different types of cancers has been published and it was shown that several miRNAs are inappropriately expressed in different cancers. Among the mechanisms that can cause this lack of proper expression are epigenetics, chromosomal changes, polymorphisms or defects in processing proteins. Recent research shows that phytochemicals, including epigallocatechin-3-gallate (EGCG), exert important epigenetic-based anticancer effects such as pro-apoptotic or anti proliferative through miRNA gene silencing. Given that EGCG is able to modulate a variety of cancer-related process i.e., angiogenesis, proliferation, metastasis and apoptosis via targeting various miRNAs such as let-7, miR-16, and miR-210. The discovery of new miRNAs and the differences observed in their expression when exposed to EGCG provides evidence that targeting these miRNAs may be beneficial as a form of treatment. In this review, we aim to provide an overview, based on current knowledge, on how phytochemicals, including epigallocatechin-3-gallate, can be considered as potential miRNAs modulator to improve efficacy of current cancer treatments.

## Introduction

The World Health Organization's Global Cancer Report suggests that, in addition to breast and lung cancer posing a considerable risk to human life and health, we may also be seeing an upswing in the number of gastric, colorectal, liver, and prostate cancer cases. It is predicted that by 2040, the global burden of cancer will be 50% higher than it is today, with 30 million new cases annually [1–3]. Although there are various treatments available for cancer, the results are far from satisfactory [4]. Chemotherapy drugs such as cisplatin, doxorubicin and even radiotherapy are frequently used in the management of many cancer cases, often causing negative side effects and severe toxicity. Radiotherapy furthermore has the risk of leading to impairments in memory, learning, and cognitive abilities, and may result in decreased brain functioning later in life [5]. In addition to the risk of developing a secondary tumor due to chemotherapy, there are also potential side effects resulting from damage to healthy tissues. In response to this, plant-derived compounds, such as alkaloids, terpenoids, phenols, flavonoids, and more, have proven to be effective in preventing and treating certain cancers, such as breast cancer lung cancer, and ovarian cancer. These compounds influence the inflammatory processes, cause cell apoptosis, inhibit the invasion and metastasis of cancer cells, and reduce the immune system's tolerance of tumors [6].

The beneficial effects of green tea are due to the presence of polyphenol compounds in it. Epigallocatechin and epigallocatechin 3-gallate are the most important and abundant polyphenols in green tea that many studies have shown to be responsible for various biological effects such as anti-methylation of DNA, anti-apoptotic, anti- inflammatory, anti-oxidant, anti-angiogenesis and anti-metastatic by modulating signaling pathways. Epigenetic effects of green tea by modulating of microRNAs involved in numerous signaling pathway has created a glimmer of hope in chemoprevention and treatment of several cancers.

MicroRNAs are small non-coding RNA molecules, ranging from 18 to 25 nucleotides in length, which are known to be involved in the regulation of gene expression. They participate in various cellular signaling pathways, such as those related to cell growth, proliferation, differentiation, survival, and apoptosis. EGCG has been demonstrated to be effective in controlling various diseases including cancer, by altering the expression of microRNAs (miRNAs). A study demonstrated that EGCG is known to influence miRNA expression in cancer, with the miRNA Let-7 being specifically up-regulated by the laminin receptor upon the compound's activation [7]. EGCG increases the production of miR-210, which has a regulating effect on the transcription factor HIF-1α, resulting in decreased progression of lung cancer [8]. Research has shown that EGCG modulating miR-16 can lead to a lower expression of the Bcl-2 protein that has an apoptotic effect in hepatocellular carcinoma. Additionally, EGCG modulation of miRNAs such as miR-15 has been linked with decreased cancerous growth and invasion in breast cancer [9]. A significant proportion of breast cancer cases with expressed hormones receptors are inhibited by miRNAs that have been modulated by EGCG [10]. Analyses of miRNA levels from cancer cells and those treated with EGCG provide a greater understanding of the involvement of miRNA in the development of cancer. A next-generation sequencing study that compared A549 lung cancer cells with A549 lung cancer cells treated with EGCG revealed that EGCG can regulate a variety of carcinogenic pathways through changes in the miRNA expression levels [11]. Herein, we highlighted potential role of EGCG against several cancers by modulating of microRNAs in various cancers in this literature review.

### Structure of EGCG

**Fig. 1:**
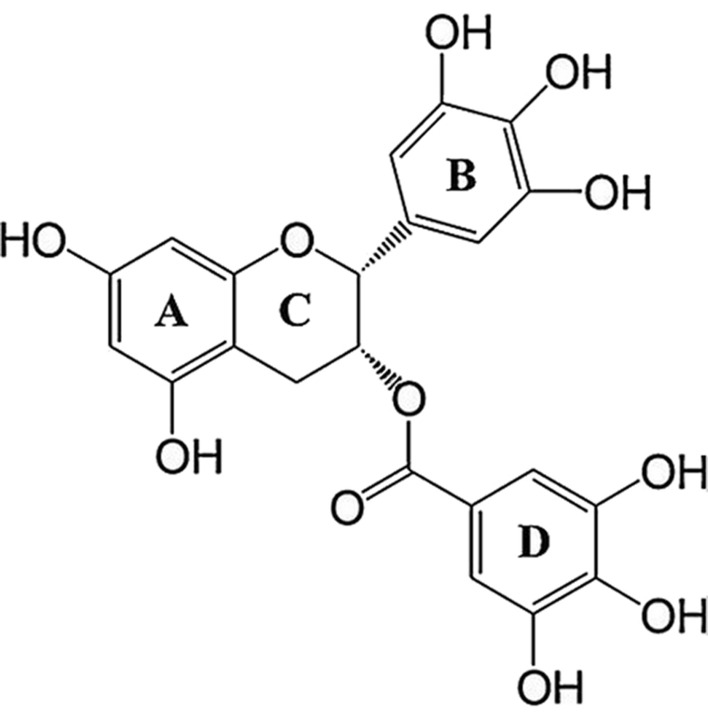
The structure of EGCG

EGCG consists of three aromatic rings (A, B, and D) which are connected by a pyran-ring (C; Fig. 1). This particular structure is thought to be the cause of EGCG's health-promoting properties. It has been suggested that its antioxidant activity is due in part to processes such as the transfer of hydrogen atoms or the transfer of single electrons, which take place among the hydroxyl groups located on the B and/or D rings [12]. Additionally, in vitro studies have indicated that both the B and D rings are linked to a reduction of proteasome function [13]. A ring of EGCG plays a part in restraining Heat-Shock Protein 90 (HSP90) [14]. The hydroxyl group on the B ring at the 5′ position has been found to impede the growth of Helicobacter pylori in the stomach [15].

### EGCG and cancer

**Fig. 2:**
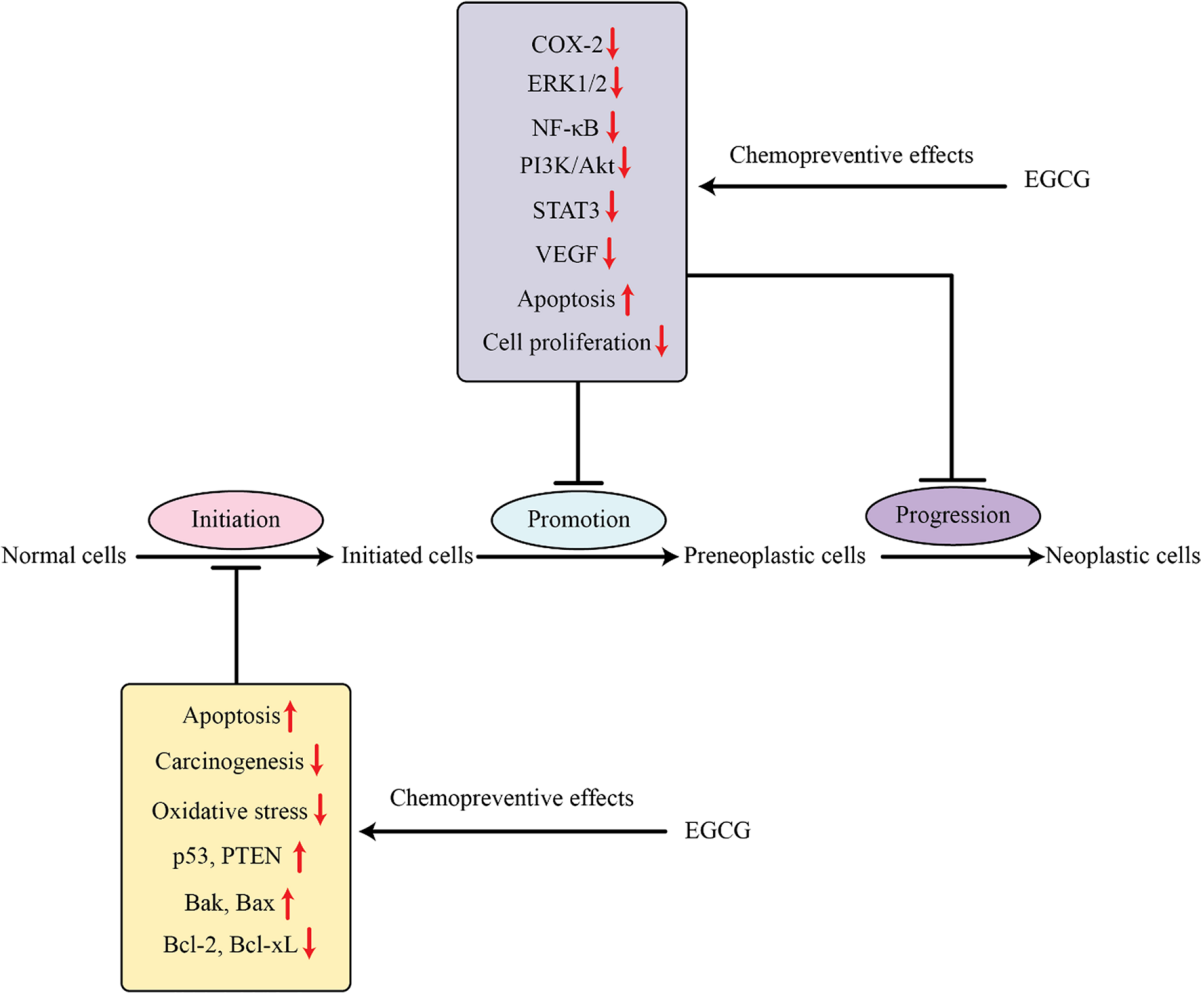
EGCG has been shown to influence the initiation, promotion, and progression of cancer by modifying multiple processes or genes. It is therefore thought to potentially play a role in the prevention and inhibition of certain cancers

Research has been conducted to evaluate the potential of EGCG to act as a chemoprevention agent on breast tumors, showing excellent antitumor effects despite issues of bioavailability being a potential cause of conflicting and uneven results [9]. EGCG has been shown to have an effect on the viability and self-repair ability of triplenegative breast cancer (TNBC) cells, making them responsive to estrogen by activating ER-α. But the exact mechanism by which EGCG works on TNBC cells is still uncertain. Recent studies have uncovered that WISP-2/ CCN5 can have an impact on the survivability, expression of ER-α, and stem cell-like qualities found in TNBC and additional forms of cancer [16]. EGCG enhances the activity of CCN5 by improving its bioavailability and enhancing its anti-tumor properties. The action of EGCG against CCN5 results in the decrease of cell viability, diminishment of sphere-forming ability and suppression of tumor growth in vivo in TNBC cells [17, 18]. EGCG plays a crucial role in the advancement of cutting-edge epigenetic-based therapy [19]. The expression of genes associated with angiogenesis was assessed to determine the anti-angiogenic effects of EGCG treatment in cancer cells. EGCG treatment was observed to reduce the regulation of genes driving invasion, adhesion, and proliferation, as well as the expression of genes identified to have antagonistic effects [20]. EGCG demonstrated a major role in suppressing melanoma cell survival and prevented NF-κB activity, which consequently led to a decrease in IL-1β production in melanoma cells [21]. ECGC was found to inhibit tyrosine phosphorylation of the focal adhesion kinase and significantly reduce the activity of MMP-9. In addition, studies on animals showed that EGCG alone was able to reduce lung metastases [22]. Despite the presence of cancerous cells, EGCG was found to be effective at halting their invasion. This inhibition was possibly related to an increase in the level of E-cadherin which would regulate the process [23]. EGCG has been demonstrated to possess anti-tumor properties through its influence on different signalling pathways [24]. EGCG has been linked to the prevention of cancer development through its inhibition of the interleukin signaling pathways, TNF and COX. This inhibition is observed to enhance the activation of the proteins PTEN/p21 and p53, control cell death via Bcl-2/Bax, and disruption of transcription factors and regulatory molecules implicated in tumor growth [25]. The action of EGCG in cancer prevention is achieved by altering genes and associated signaling molecules, leading to the prevention of the beginning, promotion, and advancement of cancer (Fig. 2). NF-κB plays an important role in tumor development by regulating genes connected to the main features of tumors, such as survival, proliferation, metastasis, and inflammation [26]. EGCG suppresses NF-κB signaling by preventing phosphorylation and therefore destruction of IκBα, which stops the nuclear translocation of the proteins p50 and p65 [27, 28]. ECGC reduces the NF-κB pathway to inhibit the growth and decrease the ability of cells to invade [26, 28, 29]. EGCG has the ability to reduce the proliferation and invasiveness of breast tumors by blocking the Wnt pathway with the help of HBP1 gene. It does this by causing deregulation of the Wnt pathway which results in increased expression of G1 regulators, c-MYC, and cyclin D1 genes. This effect also leads to a reduction in the potential of the tumor to be invasive and migratory [30]. EGCG inhibits NFκ-B and HIF-1 activation, as well as VEGF expression, inhibiting the growth of tumors and the development of breast cancer. Treatment with EGCG resulted in a decreased cancer weight relative to the control and a lower expression of VEGF [31, 32]. The anti-angiogenic and anti-proliferative properties of EGCG make it a potentially valuable therapeutic treatment for breast cancer and head and neck squamous cell carcinomas (HNSCC). It does so by limiting the activation of STAT3 and NFκ-B in cancerous cells, which prevents the production of VEGF [33]. EGCG was demonstrated to reduce VEGF-induced DNA synthesis, cell proliferation, and the self-activation process of VEGFR-1 and -2 [34]. Although tea polyphenols have a good safety profile and are considered safe when consumed in high quantities (600–1800 mg/day), there have been reports of potential toxicity related to one of the substances within that group, EGCG [35]. Schmidt and their colleagues established that EGCG plays an integral role in the toxic effects of green tea extracts on hepatocytes [36]. Additionally, supplementation of EGCG was demonstrated to worsen the harm inflicted on beta cells in diabetic rats due to high-glucose levels [37]. EGCG induces a reduction in both the number of islet cells and the amount of insulin-producing beta cells by creating reactive oxygen species (ROS) at small concentrations in the plasma [37]. There have been a lot of research done on the potentially hazardous effects of EGCG causing liver failure [38, 39].

Table 1 presents a compilation of research focusing on the impacts of EGCG on various types of cancer.

**Table 1:** The effects of EGCG in different cancers

Cancer	Dose (s)	Mechanism	Model (In vitro/ In vivo)	Cell line	Refs.
Glioma	82 and 134 μg/mL	Decrease the guidance of axon process and different metabolic-related pathways	In vitro	1321N1	[40]
Ovarian cancer	5–80 μg/mL	-Increasing the activity of Bax and caspase-3 -Decreasing the activity of Bcl-2	In vitro	NIH-OVCAR-3, SKOV3, and CAOV-3	[41]
Gastric cancer	25, 50, 100, 200, 400, 800 μg/ml	Decrease HBV infection	In vitro	HepG2.2.15	[42]
Gastric cancer	12.5, 25, 50, 100, 200 μM	Increase autophagy	In vitro	HepG2, HepG2.2.15	[43]
Gastric cancer	0–100 μg/ml	Decrease proliferation and increase apoptosis	In vitro	HepG2	[44]
Gastric cancer	0–150 μM	Increase autophagy	In vitro	HepG2	[45]
Breast cancer	5 -20 μg/mL	Increase the control of caspase-9, caspase-3, PARP	In vitro	MCF-7	[9]
Breast Cancer	5 μM	The suppression of N-cadherin	In vitro	HCC1806, MDA-MB-231, MDA-MB-157, MCF-7,	[46]
Endometrial cancer	20- 60 μM	Reduce the activity of Akt/ PI3K/mTOR/HIF-1α pathway to inhibit control of HIF-1α/VEGFA	In vitro	AN3CA, PHES, THP-1, RL95-2,	[47]
Breast cancer	10–320 μM	Increased control of caspase-9, caspase-8, caspase-3	In vitro	4 T1	[48]
Breast cancer	20–120 μmol/L	Decreasing the activity of the p53 /Bcl-2 pathway	In vitro	MCF-7	[49]
Breast cancer	0–80 μM	Reduce control of the PI3K /Akt pathway	In vitro	T47D	[50]
Breast cancer	40 nmol	Focus on pathways that either promote vascular growth or programmed cell death	In vitro	Hs578T	[51]
Breast cancer	25- 100 mg/L	Reduce the control of VEGF and HIF-1α	In vitro	MCF-7	[52]
Breast cancer	10- 50 ug/mL	Decrease control of HIF-1α, NF-κB	In vitro	MCF-7, E0771 and MDA-MB- 231	[31]
Ovarian cancer	20–100 μg/ mL	Reduce control of AQP5, NF-κB, IκB-α and p65	In vitro	SKOV3	[27]
Endometrial cancer	100 μM	The blocking of MAPK and Akt pathways	In vitro	Ishikawa cells	[53]
Breast cancer	5–20 μM	Reducing the activity of the ERK/NF-κB/PI3K pathway	In vitro	MCF-7	[54]
Breast cancer	25–100 μM	The Wnt pathway and its target gene c-MYC can be suppressed	In vitro	MDA-MB-231	[30]
Ovarian cancer	20–40 μmol/L	Lessen the control of ETAR-influenced processes	In vitro	HEY and OVCA 433	[55]
Ovarian cancer	25- 100 μM	The expression of p21 can be increased, while the expression of PCNA and Bcl-xL can be reduced, and Bax can be elevated	In vitro	SKOV-3, OVCAR-3, PA-1	[56]
Ovarian cancer	50 mg/kg	Prevents the development of cancer by controlling the activity of PTEN/mTOR/Akt pathway	In vivo	–	[41]
Glioma	87 mg/kg	Induce apoptosis	In vivo, In vitro	C6	[57]
Breast cancer	300 μg	DNMT2 methylation activity is inhibited	In vivo	–	[58]
Breast cancer	50–100 mg/ kg	Suppresses cancer VEGF expression	In vivo	–	[31]
Endometrial cancer	50 mg/kg	Preventing cancer angiogenesis and growth	In vivo	–	[59]
Endometrial cancer	65 mg/kg	The suppression of VEGF has been demonstrated	In vivo	–	[60]
Ovarian cancer	12.4 g/L	Reducing the amount of ETAR and ET-1 in cancer cells has been shown to impede their growth	In vivo	–	[55]

### EGCG and epigenetic modification

Cancer is affected by both genetic and epigenetic processes. Epigenetics can modify the gene expression without changing the genetic code, and the epigenetic mechanisms implicated in this alteration are DNA methylation and histone acetylation. These alterations lead to the formation of malignant cells. DNA methylation is the most studied epigenetic modification in human cells and is regulated by DNMTs. When DNA is hypermethylated by DNMTs, it can prevent binding of transcription factors to promoters and activate silencing proteins, resulting in gene silencing. The natural compound EGCG can interact with the catalytic site of DNMTs and inhibit its activity [61]. EGCG is capable of reversing methylation-associated decreases in the expression of the tumor suppressor the retinoic acid receptor β, p16INK4a, the DNA mismatch repair gene human mutL homolog 1 and O6-methylguanine methyltransferase, in esophageal cells, and subsequently attenuates cell growth and colony formation [62]. EGCG has been found to prevent cell growth and increase apoptosis in renal carcinoma cells, likely due to the fact that it boosts tissue factor pathway inhibitor-2 (TFPI-2) production. Since higher levels of TFPI-2 are correlated with lower levels of malignancy, this could explain why EGCG might have this effect [63]. Compared to the process of methylation, increasing histone acetylation leads to an open chromatin structure that triggers transcriptional activation. In skin cancer cells, scientists have discovered that EGCG can increase the level of acetylation on histone H3 and H4, thereby raising the expression of tumor-suppressor genes, including p16INK4a and Cip1/p21 [64]. EGCG has been shown to decrease androgen receptor acetylation, ultimately leading to the repressing of androgen-mediated transcription and cell growth in prostate carcinoma cells [65]. Recently, Ko et al. [66] indicated that EGCG could reduce Smad signalling through inhibiting acetylation in lung carcinoma cells. However, the influence of EGCG on acetylation is debatable and varies depending on the cell type and environment.

### Biogenesis and the role of miRNAs

**Fig. 3:**
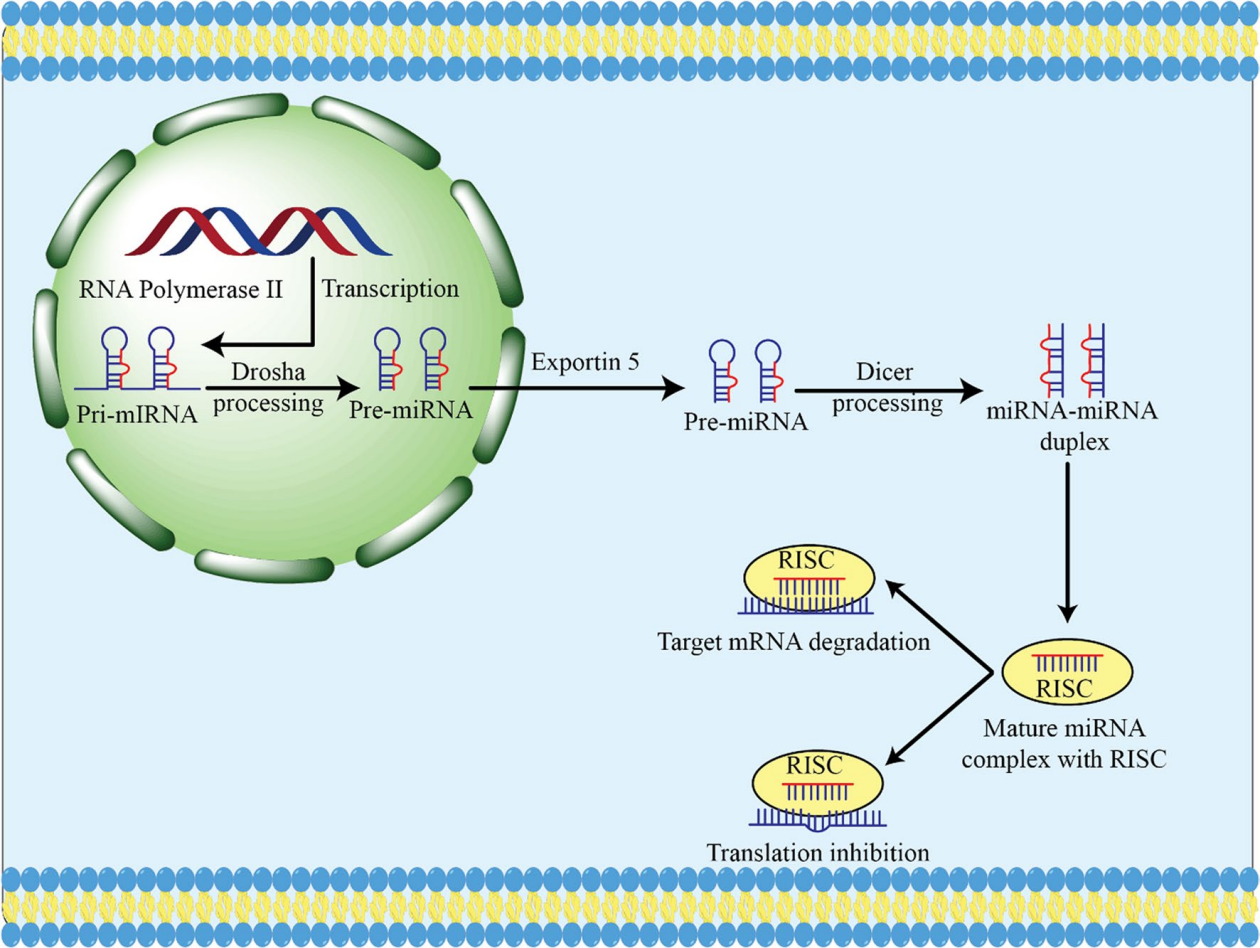
A diagrammatic summary of the standard process of miRNA creation

The majority of genes responsible for encoding miRNAs are situated in intergenic locations, while a few are in intragenic regions. Generally, miRNA transcription is done utilizing RNA polymerase II; however, there is a subsample of miRNAs located next to repetitive sequences such as Alu that are transcribed using RNA polymerase III (Fig. 3). The end product of transcription for both enzymes is the same, which is known as primary microRNA, which is denoted by a cap and a polyadenylated tail [67, 68]. The initial processing of miRNA takes place in the nucleus, wherein the pri-microRNA is cut by Drosha and its associated protein, DGCR8, at the hairpin region. This yields a stem-loop structure of roughly 70 nucleotides, known as pre-miR, which is then transported to the cytoplasm and further processed by exportin. After pre-miRNA is generated in the cytoplasm, Dicer launches the subsequent stage of processing. This enzyme leads to a double stranded molecule made of 18–25 nucleotides that consists of complimentary trailer and leader strands. The strand attaching to the Argonature proteins are destroyed, while its counterpart is then accumulated by the RNA-induced silencing complex (RISC) [69]. This evidence suggests that the 5-ends of both strands of the duplex are relatively unstable, and thus are more likely to be cleaved. However, the overall stability of the duplex has shown to remain intact, indicating that neither strand will be completely broken down [69].

### The role of miRNAs in different cancers

The first studies linking miRNAs to cancer were done in 2002 on chronic lymphocytic leukemia patients. Calin and colleagues observed that in about 65 percent of these patients, the clusters of miR-15a and miR-1-16 genes are negatively regulated or deleted [70]. In fact, their tumor suppressor role was proposed. Subsequently, the same group prepared a map of a significant percentage of miRs and they saw that 52.5 percent of them are located in areas adjacent to cancer or fragile. In the year 2005, the first report stating the lack of proper regulation of 29 miRNAs in breast cancer was published. MiRNAs expression patterns are highly regulated. These molecules have important roles in proliferation, apoptosis and differentiation. Several studies have shown that the expression profile of miRNAs in normal tissues is different from tumor tissues and it is also different between types of tumors. MicroRNAs are one of the main regulators of programmed death in the tumorigenic process, and the survival of cancer cells is controlled by manipulating these microRNAs. On the other hand, cancer cells maintain their immortality by maintaining telomeres through the upregulation of telomerase. MiRNAs that are not functioning properly can lead to an increase in telomerase activity in tumors [71]. A number of miRNA have been found to be involved in epithelial-mesenchymal transition (EMT), which is a fundamental element of the process of invasion and metastasis of tumors [72]. It is remarkable to observe that a large proportion of microRNAs are positioned at regions of instability within the genome, many of which are connected to various forms of cancer. MicroRNAs play an important role in regulating anti-cancer immune responses via modulating of antigen processing and presentation, HLA-G expression, NKG2D ligands, PD-L1 level and metabolism in cancer cells [73]. Many body of MiRNAs have been identified that can induce angiogenesis via modulating different signaling pathways such as HIPK2, PML/Smad 1/5/8, PI3K/AKT, SRCIN1, TSP-1 and KLF2/KLF4/VEGF.

### MiRNAs act as oncogenes

Studies have shown that miRNAs can have both oncogenic and tumor suppressor roles and they are called Oncomir and Ts-mir respectively. Oncomirs are observed in a range of tissue cancers and tend to occur in places of a genome with deletions, duplications, or genetic mutations. For example, 155 miRNAs are the only miRNAs that alone can induce tumorigenesis. Studies have shown that transgenic mice with increased expression of 155 miRNAs are prone to lymphoma [74]. There is enhanced expression of miRNA-155 found in a lot of different B-cell malignancies such as Hodgkin's lymphoma, a type of intense CLL and some Burkitt lymphoma. An oncogenic miRNA which is an earlier discovery is miRNA-17- 92 cluster which is located in chromosomal locus 13q31.3 in humans and it is formed from one polycistronic transcript, made up of seven microRNAs which contain miRNA-18a, miRNA-19b-1, miRNA-92-1, miRNA-17-5p, miRNA-19a, miRNA-17-3p, and miRNA-20a [75]. expression of miRNA-17-9 cluster increase in different types of cancers including lymphomas and lung cancer and many other cancers [75].

### MiRNAs act as tumor suppressor

Let-7 is a prominent miRNA whose expression is notably decreased in malignant cells. It has the notable capability to act as a tumor suppressor, repressing oncogenes or genes that prevent cell differentiation or cause cell death. Low expression of let-7 has been seen in lung tumors in comparison to normal lung tissue. Furthermore, its compulsion to express has been seen to inhibit the development of cancer cells in vivo as well as in vitro. Additionally, a reduction in the expression of let-7 is connected with patients having shorter post-surgery life spans in a variety of cancer types [76].

### Natural product compounds and miRNAs in cancer

Natural product compounds and their derivatives have been employed in the treatment of various illnesses like diabetes, neurological issues, gastrointestinal diseases, obesity, and cancer. These kinds of compounds have been praised due to their unique molecular structures, possible biological activity, and reduced harm to healthy cells, making them a valuable tool in the fight against cancer by targeting multiple cellular pathways [77]. The significant variety of natural products, coupled with their intricate mechanisms, poses a hurdle to their use in different contexts [78, 79]. Natural products have an advantage in treating tumorigenesis because their multifunctionality mechanism allows them to interact with multiple cellular signaling pathways that are involved in the multi-stage process [80, 81]. It is believed that utilizing natural products to treat cancer is more effective compared to single chemotherapy treatments because they can target multiple areas at the same time. This is why single chemotherapy is often not successful in treating cancer and why natural products may be better suited for the task [82].

**Fig. 4:**
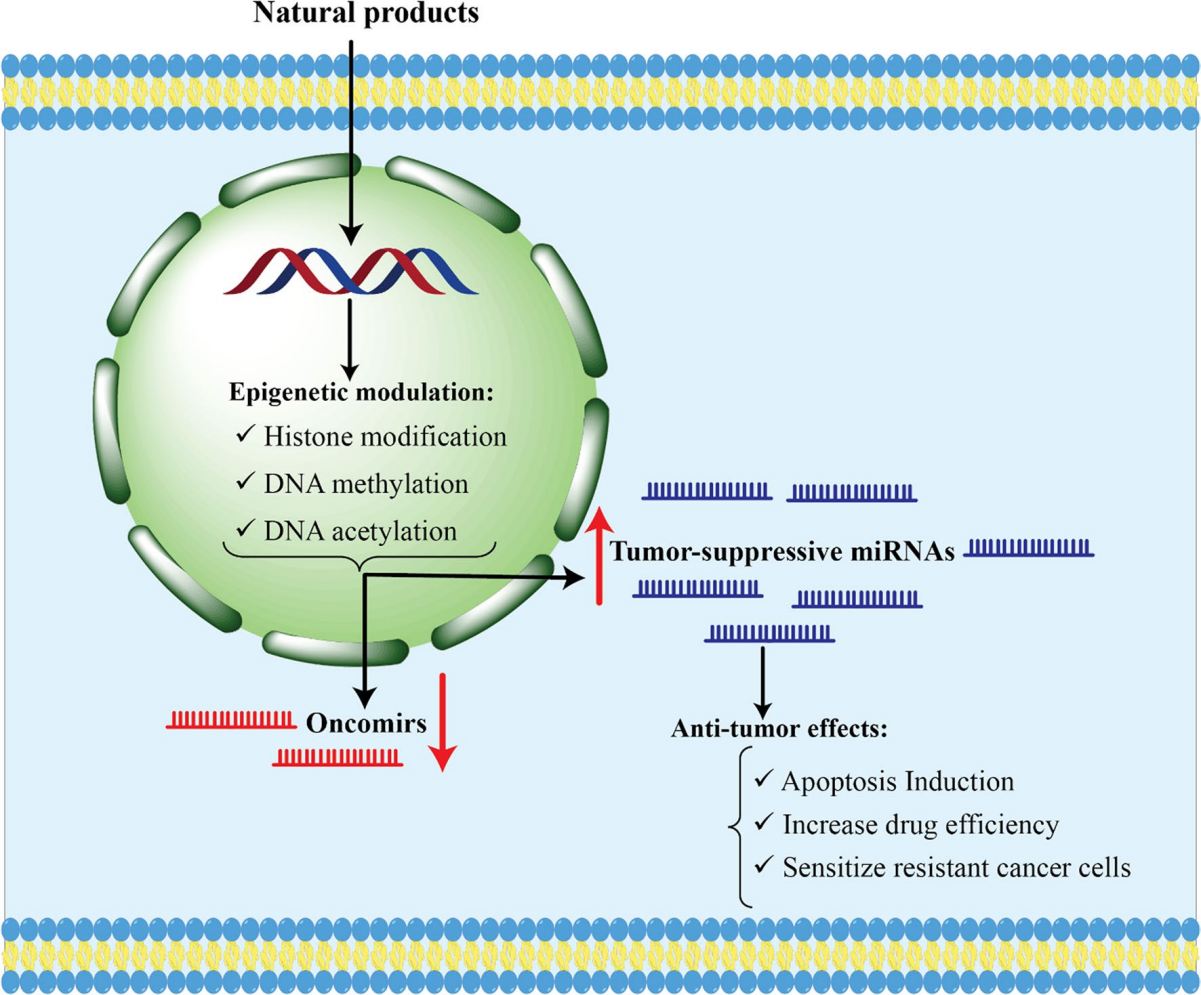
A visual representation of how natural product compounds can alter miRNAs in cancer cells

Recent evidence has shown that natural products can act against cancer growth by altering the epigenetics of cancer cells, in addition to displaying other possible biological activities [83]. Natural compounds have the capability of preventing cancer by interacting with a variety of misaligned cell signaling pathways [84]. Likewise, miRNAs influence a large number of different biological processes, including tumor growth, advancement, and cellular death processes [85]. It has been suggested that natural products have the capacity to modulate miRNAs, which could ultimately lead to therapeutic treatments for cancer and a variety of other diseases. Evidence points to the fact that both natural compounds and miRNAs affect many cellular targets, as shown in Fig. 4. The results of the research suggested that natural products can have anti-cancer properties by altering miRNA profiles, yet the exact mechanism remains unknown.

Curcumin has been found to reduce tumor cell invasion and inhibit EMT in breast cancer cells by regulating expression of miR-34a, resulting in the suppression of the CD24 Slug, and Axl, genes which are responsible for the promotion of EMT [86]. In addition, curcumin inhibits the proliferation and movement of thymic carcinoma cells, and disrupts the signaling pathways of Notch and mTOR by decreasing the expression of miR-27a [87]. Despite this, combining the MAPK inhibitor PD98059 resulted in a marked increase in the anti-tumor effects [88]. Resveratrol has an effect on multiple tumor suppressor microRNAs (miR-200c-3p, miR-409-3p, miR-542-3p, miR-122-5p, miR-125b,) to regulate the cell cycle and apoptosis of breast cancer cells. This increases the amount of Bcl-2, XIAP, and CDKs thus suppressing tumor development and inducing cell death [89].

Quercetin has been found to increase the expression of miR-16 and HOXA10, which in turn diminishes the viability of the oral cancer cells as well as the invasion, migration, and activity of MMP-2 and MMP-9 [90]. Additionally, quercetin has been found to have an anti-cancer effect in OSCC by increasing the level of miR-22. It is believed that by raising miR-22 with the assistance of quercetin, levels of Wnt1/β-catenin signaling are diminished, both in lab and animal testings, resulting in a reduced tumor mass [91].

### Epigallocatechin-3-gallate and the modulation of miRNAs in different cancers

Green tea produced from the leaves of the Camellia sinensis plant is a very popular drink around the world. Green tea polyphenols have preventive effects due to their anticancer properties in many cancers, including breast cancer [49], hepatocellular carcinoma (HCC) [92], prostate cancer [93], gastric and colorectal cancer [94], Lung cancer [95], and are also useful in the treatment of diabetes, Parkinson's, stroke, Alzheimer's and obesity. Important polyphenols of green tea are Epicatechin (EC), (Epigallocatechin) (EGC), (Epicatechin-3-gallate) (ECG) (Epigallocatechin-3-gallate) (EGCG). J Qin's study with the effect of EGCG on the T24 bladder cancer cell line resulted in beneficial results, including the inhibition of cancer cell growth by the effect of EGCG[96]. Jun Ma and his colleagues evaluated cell survival and death in the treatment of AGS gastric cancer cells with EGCG (a component of green tea) and stated that EGCG contained in green tea extract promotes apoptosis and inhibition of proliferation in AGS gastric cancer cells [97]. In another study, Nishikawa performed EGCG treatment on hepatocellular carcinoma cell line (HCC) and reported that EGCG inhibited the growth of these cancer cells and also decreased the expression of Bcl_2 and Bcl_XL genes as its concentration increased [98]. Green tea' catechins anticancer effects may be caused by a number of different methods, including inhibition protein synthesis, lipogenesis, cell growth cell motility and invasion, telomerase, angiogenesis, induction apoptosis and cell death. It seems that the chemotherapeutic effects of catechins take place through the regulation of microRNA expression profile. According to a study, treatment of different cervical carcinoma cells to EGCG showed that can inhibit cell growth and induce apoptosis via modulating various miRNAs expression [99]. The expression of hsa-miR-221, hsa-miR-222, hsa-miR-21, hsa-miR-146b and hsa-miR-204 involved in thyroid cancer progression, differentiation Thyroid-specific genes in the anaplastic thyroid carcinoma (ATC) cell lines (8505C cell line and SW1736 cell line) was assessed after treatments with EGCG in another study. EGCG increased all the mRNAs related to differentiation Thyroid-specific genes in 8505C cells and reduced in SW1736 cells. In SW1736, EGCG had no effect on cell growth ability, while it had a reducing effect on the formation of colonies in 8505C cells.in this study, only hsa-miR-221 downregulated by EGCG and had no effect on the expression of other miRNAs involved in tumor tumorigenesis [100]. Appari et al. [101] attempted to improve promising pancreatic cancer therapy by single or combined Sulforaphane, quercetin and catechins. In MIA-PaCa2 cells, single bioactives significantly reduced the colony-forming capacity and effect of ECG or CG was superior to EGCG. The combination quercetin/sulforaphane and sulforaphane/EGCG had more strong effect in inhibition colony formation than single bioactives. Besides, in MIA-PaCa2 and BxPc-3 cells, the effect of the combination of natural bioactives in reducing spheroid formation, viability and apoptosis was stronger compared to single doses. Reduced expression of miR-let-7 and increased expression of K-ras in different cancers is well proven. Therefore, in this study, effect of treatment of combination or single natural bioactives on expression of miR-let-7 and K-ras assessed in MIA-PaCa2, BxPC-3, primary PDA cells, PacaDD-183 and CRL 1097 non-malignant pancreatic ductal cells. Single or combination treatments had strong effects on up-regulation of miR-let-7 and down-regulation of K-ras in all cells expect non-malignant CRL 1097 cells [101]. Next-Generation Sequencing (NGS) study using MDA-MB-231 cells and EGCG-treated MDA-MB-231 cells showed that EGCG can harmonize different breast cancer-related pathways by modifying 873 known and 47 novel miRNAs expressions. This study, potential role of EGCG in up- and down-regulation of tumor suppressor and oncogenic miRNAs, respectively is well confirm [102]. In another study, Green Tea’ EGC and EGCG alone significantly altered the key molecules in neuroblastoma SH-SY5Y and SK-N-DZ Cells that have role in extrinsic and intrinsic apoptotic pathways and induced morphological features of apoptosis in these cells [103]. miR-7-1 expression in both cell lines increased via treatment with EGC or EGCG. This microRNA is a potent tumor suppressor that cells committed to entry apoptosis process [103]. Table 2 lists various studies on the effects of EGCG on microRNAs in various cancers.

**Table 2:** The effects of EGCG on microRNAs in various cancers

microRNA	Study type	Model	Target	Effects	Refs.
miR-21, miR-16	In vitro	Breast cancer	Down	Controlling NF-kB	[104]
hsa-miR-204, hsa-miR-222, hsa-miR-21, hsa-miR-146b, hsa-miR-221	In vitro	Thyroid cancer	Up	Improving the apoptosis	[100]
miR-106b, miR-93, miR-92	In vitro	Malignant neuroblastoma	Down	Improving the apoptosis	[105]
miR-99a, miR-34a, miR-7-1	In vitro	Malignant neuroblastoma	Up	Improving the apoptosis	[105]
miR-27, miR-21	In vitro	Breast cancer	Dow	Changes in the miRNA control of possible cancer-causing genes and tumor-suppressing genes	[10]
miR-16	In vivo	Breast cancer	Up	NF-κB pathway	[106]
miR-485	In vivo	Non-small cell lung cancer	Up	Control miR-485/CD44 axis	[107]
hsa-mir-485-5p	In vivo	Non-small cell lung cancer	Up	Control the hsa-mir-485-5p/RXRα axis	[108]
miR483-3p	In vivo	Hepatocellular carcinoma cells	Down	Hypermethylation of the miR483-3p promoter region	[109]
miR-155-5p	In vitro	Colorectal Cancer	Up	Suppressing GRP78/NF-κB/miR-155-5p/MDR1 Pathway	[110]
miR-1226-3p, miR-185-3p, miR-642a-5p, miR-3116, miR-31-5p,	In vitro	Bladder cancer	Up	Modulation Hippo signaling pathway	[111]
hsa-miR-1915 hsa-miR-29b-1-5p hsa-miR-210 hsa-miR-1225-5p hsa-miR-1202 hsa-miR-1246 hsa-miR-1973 hsa-miR-3162 hsa-miR-4281 hsa-miR-3656 hsa-miR-3665 hsa-miR-1207-5p hsa-miR-3196 hsa-miR-34a hsa-miR-2861	In vitro	Nasopharyngeal carcinoma	Up	Apoptosis, Cell cycle, Cell proliferation	[112]
miR-296	In vitro	Nasopharyngeal Carcinoma	Up	Suppressing migratory properties of anoikis-resistant cells	[113]
miR-34a, let-7a	In vitro	Hepatocellular carcinoma	Up	Improving cytotoxicity and inducing the apoptotic pathway	[114]
miR-195	In vitro	Prostate cancer	Up	Affect on EMT	[115]
miR-181a	In vitro	Prostate Cancer	Up	Prompting the cellular apoptosis	[116]
miRNA-21	In vivo	Prostate cancer	Down	Suppressing cells growth and Antagonist of AR signaling and	[117]
miRNA-330	In vivo	Prostate cancer	Up	Suppressing cells growth and antagonist of AR signaling and	[117]
miR-200c, miR-145, miR-34a,	In vitro	Colorectal cancer	Up	Suppressing Notch and Bmi1, Ezh2, and Suz12	[118]
miR-210	In vitro	Lung cancer	Up	Anti-proliferation	[8]
miR-30e-3p	In vitro	Malignant melanoma	Up	Controlling circ_MITF/miR-30e-3p/HDAC2 axis	[119]
microRNA-let-7b	In vitro	Malignant melanoma	Up	Down-modulstion of high mobility group A2 (HMGA2)	[120]
miR-204	In vitro	Oral squamous cell carcinomas	Down	miR204-mediated inhibition of Sox4 and Slug	[121]
miR-25	In vitro	Breast cancer	Down	Anti-proliferation and pro-apoptosis	[9]
let-7	In vitro	Lung cancer	Up	Let-7 signaling pathway	[122]
hsa-miR-98-5p	In vitro	Non-small cell lung cancer	Down	Elevating of p53	[123]
miR-133a/b	In vivo	Prostatic hyperplasia	Down		[124]
miR-203, miR-125b	In vitro	Cervical carcinoma	Down	Induce cell cycle arrest and apoptosis	[99]
miR-29a, miR-210, miR-29, miR-203, miR-125b,	In vitro	Cervical carcinoma	Up	Induce cell cycle arrest and apoptosis	[99]

## Data Availability

Not applicable.
